# Broadly Neutralizing Human Anti-HIV Antibody 2G12 Is Effective in Protection against Mucosal SHIV Challenge Even at Low Serum Neutralizing Titers

**DOI:** 10.1371/journal.ppat.1000433

**Published:** 2009-05-15

**Authors:** Ann J. Hessell, Eva G. Rakasz, Pascal Poignard, Lars Hangartner, Gary Landucci, Donald N. Forthal, Wayne C. Koff, David I. Watkins, Dennis R. Burton

**Affiliations:** 1 Department of Immunology and Microbial Science, and IAVI Neutralizing Antibody Center, The Scripps Research Institute, La Jolla, California, United States of America; 2 Department of Pathology and Laboratory Medicine, University of Wisconsin-Madison, Madison, Wisconsin, United States of America; 3 International AIDS Vaccine Initiative (IAVI), New York, New York, United States of America; 4 Institute of Medical Virology, University of Zürich, Zürich, Switzerland; 5 Division of Infectious Diseases, Department of Medicine, UC Irvine School of Medicine, University of California Irvine, Irvine, California, United States of America; Harvard Medical School, United States of America

## Abstract

Developing an immunogen that elicits broadly neutralizing antibodies (bNAbs) is an elusive but important goal of HIV vaccine research, especially after the recent failure of the leading T cell based HIV vaccine in human efficacy trials. Even if such an immunogen can be developed, most animal model studies indicate that high serum neutralizing concentrations of bNAbs are required to provide significant benefit in typical protection experiments. One possible exception is provided by the anti-glycan bNAb 2G12, which has been reported to protect macaques against CXCR4-using SHIV challenge at relatively low serum neutralizing titers. Here, we investigated the ability of 2G12 administered intravenously (*i.v.*) to protect against vaginal challenge of rhesus macaques with the CCR5-using SHIV_SF162P3_. The results show that, at 2G12 serum neutralizing titers of the order of 1∶1 (IC_90_), 3/5 antibody-treated animals were protected with sterilizing immunity, i.e. no detectable virus replication following challenge; one animal showed a delayed and lowered primary viremia and the other animal showed a course of infection similar to 4 control animals. This result contrasts strongly with the typically high titers observed for protection by other neutralizing antibodies, including the bNAb b12. We compared b12 and 2G12 for characteristics that might explain the differences in protective ability relative to neutralizing activity. We found no evidence to suggest that 2G12 transudation to the vaginal surface was significantly superior to b12. We also observed that the ability of 2G12 to inhibit virus replication in target cells through antibody-mediated effector cell activity *in vitro* was equivalent or inferior to b12. The results raise the possibility that some epitopes on HIV may be better vaccine targets than others and support targeting the glycan shield of the envelope.

## Author Summary

An effective HIV vaccine should elicit broadly neutralizing antibodies, i.e. antibodies that neutralize a wide spectrum of different HIVs *in vitro*. A number of human monoclonal antibodies have been isolated with broad neutralization and shown to protect macaques against vaginal HIV challenge. Protection is generally correlated with neutralization and requires relatively high antibody concentrations that may be difficult to achieve by vaccination. Here, we show that one monoclonal antibody (2G12) is unusually potent in protection relative to its neutralizing ability as hinted at by earlier data. Further studies eliminate an unusual ability of 2G12 to be transported to the vagina (site of infection) as a possible explanation for our observations. Although the precise mechanism is unclear, the studies have important implications for HIV vaccine design in general by suggesting that some vaccine targets on HIV may be better than others and, specifically, by suggesting that the sugar coat of HIV may be a particularly rewarding target if appropriate immunogens can be designed.

## Introduction

There is widespread acceptance that eliciting neutralizing antibodies is likely to be an important goal of an effective HIV vaccine [1],[2],[3]. A good correlation is generally reported between the ability of an antibody to neutralize *in vitro* and to protect *in vivo* against HIV in animal models [4],[5],[6],[7],[8],[9]. The most quantitative studies have titrated the ability of specific antibodies to protect and found that sterilizing immunity is achieved when the serum concentration of antibody in the challenged animals is many multiples of the *in vitro* neutralization titer [4],[8],[10]. For instance, Nishimura, *et al.* reported that 99% of macaques were protected against intravenous challenge with an R5 SHIV_DH12_ by a specific polyclonal antibody at a 100% neutralization titer of 1∶38 [10]. In another example, we have reported sterilizing immunity against R5 SHIV_SF162P4_ vaginal challenge in 4/4 macaques with a dose of the broadly neutralizing human antibody b12 yielding a serum neutralizing titer of about 1∶400 at challenge [8]. The titer corresponded to 90% neutralization in a PBMC assay. Nishimura et al [10] estimated that this titer corresponded to 1∶32.5 or greater in their assay system providing good correspondence between the two studies. At an antibody dose giving a serum neutralizing titer of about 1∶80 in the Parren, *et al.* study, 2/4 macaques showed sterilizing immunity and the other 2 were infected with a delayed and lower primary viremia as compared to controls. At an antibody dose giving a serum neutralizing titer of about 1∶16, no animal was protected but there was a slight delay and some lowering in the magnitude of primary viremia.

Most other studies have not titrated the ability of antibodies to protect but high serum concentrations of antibody relative to neutralizing titer were generally used and shown to provide protection against virus challenge [4],[5],[6],[9],[11]. The one notable exception is provided by studies of Mascola and colleagues [7] on protection by the broadly neutralizing human MAb 2G12. In particular 2/4 macaques showed sterilizing immunity when challenged by an ×4 SHIV (SHIV_89.6P_) when the serum neutralizing titer, as measured at 90% neutralization in a PBMC assay, was less than 9. In fact, the mean concentration of 2G12 in the sera of the animals at challenge was calculated to provide 90% neutralization only with neat serum (i.e. 1∶1 neutralizing titer). The actual concentration of 2G12 in the protected animals at the time of challenge was relatively high, about 200 µg/ml following an *i.v.* administration of 15 mg/kg antibody, but 2G12 is relatively poor at neutralization of SHIV_89.6P_ (IC_90_∼200 µg/ml) hence the low neutralizing titer. The authors also carried out protection experiments with mixtures of antibodies, including 2G12. These experiments when taken together again suggested that 2G12 may provide protection that is unusually effective relative to its neutralizing titer.

Monoclonal human IgG1 2G12 is a very interesting and unique antibody. It is broadly neutralizing, particularly against clade B HIV-1 isolates [12],[13],[14]. It has a domain-exchanged structure that leads to closely proximal antibody combining sites that are well suited to the recognition of a cluster of oligomannose residues on the glycan shield of HIV [12],[15],[16],[17],[18]. 2G12 belongs to a small set of human MAbs that are described as broadly neutralizing and that recognize distinct epitopes on the HIV envelope spike. The MAb b12 recognizes an epitope overlapping the CD4 binding site “on the side” of the spike and the MAbs 2F5, 4E10 and Z13e1 recognize gp41 very close to the viral membrane, whilst 2G12 recognizes an epitope which is more on the “top” of the spike [19],[20],[21].

Given the suggestion that 2G12 may have unusual prophylactic activities and given the potential importance of this for HIV vaccine design, we decided to carry out a macaque protection study using a virus different from that of Mascola and colleagues and to pursue potential properties of 2G12 that might correlate with protection. Ideally, we would have had available a SHIV that was relatively neutralization sensitive to 2G12 to permit study of a maximum dynamic range of 2G12 concentrations with neutralizing activity. However, currently available SHIVs are relatively resistant to 2G12 and the R5 virus SHIV_SF162P3_ was chosen as the most sensitive to 2G12 neutralization. An R5 virus was thought to be more appropriate for modeling human infection than an ×4 virus. The challenge virus was used intravaginally following pre-administration of 2G12 intravenously. The results indicate that 2G12 can provide protection against an R5 virus challenge at a surprisingly low neutralization titer. Unusually efficient transport to the vaginal mucosal surface does not appear to explain the activity of 2G12. The results support targeting the glycan shield through vaccine design.

## Results

The ability of 2G12 to neutralize a panel of SHIVs in PBMC and pseudovirus assays was first assessed. A comparison with b12 was included in the study. As shown in Table 1, 2G12 was not particularly effective against any of the SHIVs tested. The activity of 2G12 against the R5 SHIV_SF162P3_ was comparable to that against the ×4 SHIV_89.6P_ used in previous studies described above and was chosen for macaque studies.

**Table 1:** Comparison of SHIV neutralization by b12 and 2G12 in rhesus PBMC-based and pseudovirus luciferase reporter gene assays.

Virus	MAb	Rhesus PBMC-based Neutralization Assay		Pseudovirus-based Neutralization Assay	
		*(µg/ml)*			
		*IC_50_*	*IC_90_*	*IC_50_*	*IC_90_*
**SHIV _89.6P_**	**2G12**	20	>900	2.6	>50
	**b12**	300	>900	11.5	>50
**SHIV _SF162P3_**	**2G12**	20	900	7.6	>50
	**b12**	2	8	0.29	2
**SHIV _BaL_**	**2G12**	>300	>300	1	>50
	**b12**	<1.2	<1.2	0.02	0.09

The selection of SHIV_SF162P3_ for the protection study was based on the results of 2G12 neutralization of rhesus PBMCs and pseudovirus assays against the panel shown.

**Figure 1:**
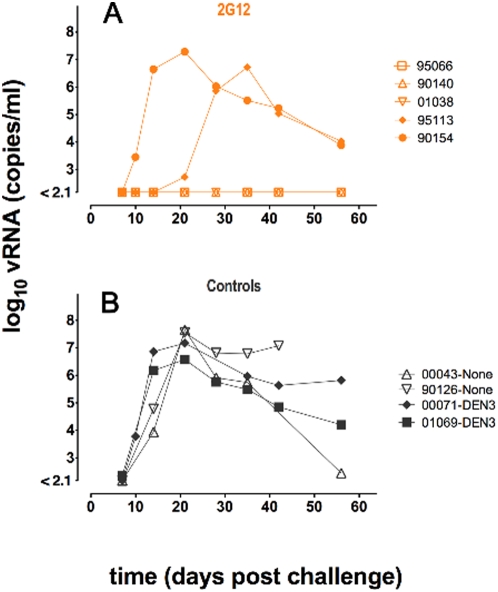
Plasma viral loads following SHIV_SF162P3_ vaginal challenge of 2G12-treated and control macaques. A total of nine female Indian rhesus macaques were divided into treatment groups of five animals for *i.v.* administration of 2G12, two animals to receive the isotype control (Dengue anti-NS1, DEN3), and two additional controls were challenged prior to the beginning of the protection study to confirm viral fitness, but were not treated with antibody. In (A) two 2G12-treated (40 mg/kg) animals became infected: 90154 reached peak viremia of 2×10^7^ on day 21 similar to controls; 95113 showed a one-week delay of infection onset and peak viremia was lower at 5×10^6^. The remaining three 2G12-treated animals were protected against infection and showed no measurable viremia. In (B) all 4 control animals experienced peak viremia between 1×10^7^ and 4×10^7^ on day 21. The quantity of SIV viral RNA genomic copy equivalents (vRNA copy Eq/ml) in EDTA-anticoagulated plasma was determined using quantitative RT PCR [52]. The assay minimum detection is 150 copies of vRNA Eq/ml (2.1 log) with a 99% confidence level.

Figure 1 depicts the outcome of the protection study that was carried out with five 2G12-treated animals, two antibody isotype (anti-Dengue NS1 IgG1, DEN3) treated control animals and two antibody-untreated control animals. The Indian rhesus macaques were first treated with Depo-Provera to thin the vaginal epithelium and to synchronize menstrual cycles [8],[22]. One day before vaginal challenge with 500 TCID_50_ (50% tissue culture infectious doses) of SHIV_SF162P3_, each animal was given an intravenous dose of 40 mg/kg of either 2G12 or the isotype control antibody. Prior to the protection experiment, two additional control animals were challenged with 500 TCID_50_ SHIV_SF162P3_ without administration of antibody to verify the infectivity of the viral stock. Blood was drawn from the animals at regular intervals following challenge to monitor viral infection, serum levels of passively administered antibody and serum neutralizing activity. The 4 control animals became infected with peak viremias of approximately 10^7^ virus copies per ml between days 14 and 21 as is generally noted in this system [9]. Two of the five 2G12-treated animals also became infected. One was infected with viral kinetics closely similar to that of the control animals. The second had a notably delayed and lower peak viremia at day 35. Three of the five 2G12-treated animals were protected and showed no detectable viremia at day 55. In order to determine whether breakthrough infection may be associated with selection of antibody escape mutants, we attempted to sequence the env gene from plasma virus of the unprotected animals. Env from animal 95113 could not be amplified but interestingly sequence analysis of animal 90154 env revealed a T388A mutation disrupting the position 386 N-glycosylation and consistent with 2G12 escape. The characterization of a 2G12 escape mutant in one of the unprotected animals raises the possibility that such variants already exist in the inoculum. Alternatively, in the presence of Ab, in particular at the suboptimal concentration achieved in the study, a certain level of viral replication may take place following challenge, allowing in some cases for the generation and selection of an escape mutant. Such scenario would suggest that the mechanism of antibody protection is not only to prevent cell infection but also to abort an already ongoing infection, presumably though effector functions as discussed below. These two possible scenarios for escape are currently under investigation.

The 2G12 antibody concentrations in the sera of the macaques at different time points were measured using three different ELISA formats. With a few exceptions, the determined serum concentrations derived from the three formats were generally in good agreement (Table 2). The first format used was an ELISA based on the ability of 2G12 to specifically recognize a relatively conserved cluster of oligomannose glycans. Serum was titrated against an immobilized synthesized oligomannose dendron conjugated to BSA [21]. The second ELISA format was based on a highly specific anti-idiotype-2G12 antibody (MIgG1 L13) that does not block the binding of gp120 or inhibit the neutralizing ability of 2G12 [23]. For comparison, a third ELISA format using monomeric gp120 _JR-FL_ was used to measure the transferred 2G12 contained in the macaque serum. In all formats, a dilution series of serum was compared to a 2G12 standard curve and the concentration determined using a non-linear regression curve fit analysis.

**Table 2:** 2G12 serum antibody concentrations following passive administration.

Protected				Not Protected			
95066	gp120	Man 4D	anti-Id	95113	gp120	Man 4D	anti-Id
Day	Serum Ab μ g/ml			Day	Serum Ab μg/ml		
**−1**	0	0	0	**−1**	0	0	0
**0**	1215	1241	911	**0**	822	1146	998
**3**	696	994	733	**3**	571	494	567
**7**	793	816	599	**7**	494	510	355
**10**	698	529	500	**10**	463	456	465
**15**	767	328	446	**15**	402	134	177
**22**	651	252	335	**22**	230	49	155

90140	gp120	Man 4D	anti-Id	90154	gp120	Man 4D	anti-Id
Day	Serum Ab μg/ml			Day	Serum Ab μg/ml		
**−1**	0	0	0	**−1**	0	0	0
**0**	954	901	1250	**0**	1252	1174	922
**3**	640	704	1005	**3**	750	575	545
**7**	694	545	838	**7**	597	371	448
**10**	640	398	705	**10**	554	221	348
**15**	453	452	502	**15**	453	174	328
**22**	173	394	505	**22**	231	101	342

1038	gp120	Man 4D	anti-Id
Day	Serum Ab μg/ml		
**−1**	0	0	0
**0**	1203	921	891
**3**	959	527	717
**7**	927	413	714
**10**	898	365	690
**15**	605	220	496
**22**	296	103	147

The concentrations of transferred 2G12 in the serum of all experimental animals on the day of challenge (day 0) and during the following three weeks were determined by ELISA using three different formats. For each animal, the results from the different ELISA formats are shown in separate columns. The concentrations of 2G12 in the macaque sera were determined from the measurement of binding to monomeric JR-FL (gp120), to an immobilized synthetic oligomannose dendron conjugated to BSA [21] (Man4D), and to a highly specific anti-idiotype-2G12 antibody (MIgG1 L13) [23] (anti-id). In all formats, a dilution series of serum was compared to a 2G12 standard curve and the concentration determined using a nonlinear regression curve fit analysis performed in GraphPad Prism Software for Mac, Version 5.0a.

The *i.v*. transfer of 2G12 at 40 mg/kg resulted in a high 2G12 serum concentration at the time of challenge that varied between 0.9 and 1.2 mg/ml. Table 3 summarizes the half-life of serum 2G12 that varied between 7.2 and 15.6 days in the 5 macaques. The average half-life of all animals as measured in the three ELISA formats is about 11 days (Table 3). The half-life of 2G12 in rhesus macaques has previously been noted as about 13 days [5].

**Table 3:** Half-life of transferred 2G12 in macaque serum.

Half-life (days) of 2G12 in macaque serum						
	Protected			Not Protected		Average (days)
Animal	95066	90140	01038	95113	90154	
**gp120 ELISA**	12.2	9.2	7.7	9.2	9.9	9.6
**Man4D ELISA**	10.7	15.6	7.2	10.5	8.4	10.5
**L13 anti-idiotype ELISA**	12.9	13.4	9.8	10.4	15.1	12.3

The data represents the half-life (t_1/2_) of serum 2G12 determined from data in three different ELISA formats over a period of three weeks following *i.v*. transfer of 40 mg/kg of 2G12. The half-life of transferred 2G12 ranged between 7.2 and 15.6 days in the 5 macaques with a somewhat shorter half-life observed in animal 01038. The average half-life of all animals as measured in the three ELISA formats is about 11 days. The half-life of 2G12 in rhesus macaques has previously been noted as about 13 days [5].

While for b12, 90% neutralization titers (IC_90_) of approximately 1∶80 in a PBMC assay were associated with protection in 50% of SHIV_SF162P4_ [8] and 90% of SHIV_SF162P3_ [9] challenged animals, a titer of only 1∶1 was sufficient to protect 60% of all animals with 2G12 in the experiment described here. It should be noted the IC_90_ for 2G12 neutralization of PBMC is approximately 900 µg/ml and the serum concentration of 2G12 at challenge was approximately 900–1,200 µg/ml making it impractical to directly measure neutralization in the PBMC assay. Using the generally more sensitive pseudovirus assay, 90% neutralization was not reached at a 1∶50 serum dilution for any of the 2G12-treated animals. Therefore, it does appear that MAb 2G12 can offer substantial protection at relatively low serum neutralizing titers.

**Figure 2:**
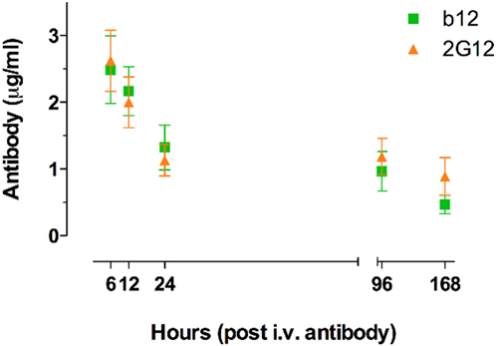
Comparison of b12 and 2G12 transudated to the vagina following intravenous administration. Each antibody treatment group consisted of three female Indian Rhesus macaques which were *i.v.*-administered 5 mg/kg of either b12 or 2G12 following Depo-provera treatment. Vaginal secretions from each animal were absorbed to cellulose wicks. A set of 3 samples per animal was taken at 6 hours, 12 hours, 24 hours, 4 days, and 7 days post *i.v.* antibody administration. The concentration of antibody in mucosal secretions was determined by ELISA from the clarified supernatant extracted from the wicks. Resulting data were compared to the corresponding antibody standard curve using nonlinear regression. Arithmetic means and standard deviations were calculated for each set of triplicate samples per animal. Data points were calculated from all animals at each timepoint and error bars represent the standard error of means. The typical time for viral challenge in protection experiments is indicated. The differences in the mean concentrations of b12 and 2G12 at each timepoint were evaluated in a student's t test and determined to be non-significant. Analyses performed in GraphPad Prism Software for Mac, Version 5.0a.

We next compared properties of 2G12 and b12 that might help explain the observed differences in protective activity relative to serum neutralization. One possibility is a gross difference in transudation efficiency for the two MAbs. For b12, it has been noted previously that the concentration of antibody at the vaginal surface following passive administration is only a small fraction of that in the serum [8]. If 2G12 was transudated to the vaginal surface much more efficiently than b12 then it is possible that it could achieve comparable neutralizing titers in vaginal fluids. This might lead to improved protection although it should be noted that no correlation between mucosal antibody levels and protection has been established. Earlier data suggests that 2G12 is not transudated unusually effectively although vaginal concentrations can vary widely [7]. We carried out a direct comparison of vaginal concentrations of b12 and 2G12 for a time period of 7 days after *i.v*. administration of 5 mg/kg MAb to 3 control macaques. As shown in Figure 2, the concentrations of the two MAbs transudated to the vaginal mucosal surface are similar and thus transudation is unlikely to contribute to protection differences between the MAbs.

**Figure 3:**
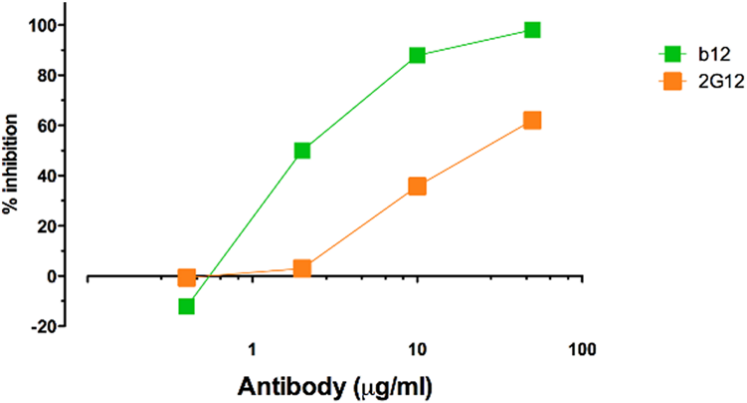
Comparison of antibody-dependent cell-mediated viral inhibition (ADCVI) by 2G12 and b12. Target cells (CEM.NKR-CCR5) were infected with SHIV_SF162P3_ and incubated for 48 hours, washed to remove cell-free virus and combined with Rhesus PBMC effector cells and serially diluted antibody. Viral inhibition was measured after incubation for 7 days. 2G12 is somewhat less effective than b12 in mediating ADCVI for a strict concentration comparison. An unpaired Two-tailed t test (P = 0.3285) of b12 and 2G12 ADCVI with an F test comparison of variance reveals no significant difference (P = 0.4154). Analysis performed in GraphPad Prism Software for Mac, Version 5.0a.

Evidence has been obtained to suggest that interaction of b12 with Fc receptors can contribute to protection against SHIV_SF162P3_ challenge in macaques [9]. In particular, it was noted that wild-type b12 mediated antibody-dependent cell-mediated virus inhibition (ADCVI) and was effective at protection, whereas a b12 variant lacking FcR binding did not mediate ADCVI and was less effective than the wild-type antibody. If 2G12 were effective at ADCVI then this might contribute to enhanced protective activity. Figure 3 suggests that 2G12 is somewhat less effective than b12 at ADCVI on the basis of a strict concentration comparison. The significance of this result is discussed further below.

Since two MHC class I alleles (Mamu-B*08 and –B*17) have been associated with elite control of SIV replication, we evaluated all experimental animals by MHC genotyping (Table 4). We used PCR-SSP to test for a panel of 9 class Ι alleles previously shown to be important in SIV epitope presentation [24],[25],[26],[27],[28]. Protected animal 90140 was positive for Mamu-A*01, an allele that appears with high frequency in many colonies and has been associated with moderate reduction of SIVmac239 replication [27],[28],[29],[30]. Protected animal 95066 expresses the Mamu-B*01 allele. This allele remains on the panel based on early reports of SIV-derived epitopes [31],[32], but studies have shown that Mamu-B*01 does not bind SIV-derived epitopes and has no effect on SIV disease progression [26]. However, even with the presence of the Mamu-A*01 allele (which is not associated with elite control of SIV replication) in animal 90140, there is no apparent correlation with the allelic profiles of the animals in this study that would account for any unusual ability to resist infection.

**Table 4:** MHC genotyping of macaques against MHC Class Ι alleles.

Animal	A01	A02	A08	A11	B01	B03	B04	B08	B17
95066^P^	−	−	−	−	+	−	−	−	−
90140^P^	+	−	−	−	−	−	−	−	−
01038^P^	−	−	−	−	−	−	−	−	−
95113^N^	−	−	−	−	−	−	−	−	−
90154^N^	−	−	−	−	−	−	−	−	−
00071^I^	−	−	−	−	−	−	−	−	−
01069^I^	−	−	−	−	+	−	−	−	−
90126^C^	−	−	−	−	+	−	−	−	−
00043^C^	+	−	−	−	−	−	−	−	−

Macaque samples were tested against a panel of nine MHC class Ι alleles that have previously been shown to be important in SIV epitope presentation or increased resistance to SIV infection [24],[25],[26]. The alleles are: Mamu-A*01, Mamu-A*02, Mamu-A*08, Mamu-A*11, Mamu-B*01, Mamu-B*03, Mamu-B*04, Mamu-B*08, and Mamu-B*17. Animal 90140 is positive for Mamu-A*01 and animal 95066 was determined to carry the Mamu-B*01 allele. Mamu-A*01 has been associated with moderate control of SIVmac239 replication [29],[30]. Mamu-B*01 remains on the panel based on early reports of SIV-derived epitopes [31],[32], but subsequent studies show that Mamu-B*01 does not bind SIV-derived epitopes and has no effect on SIV disease progression [26]. **^P, N, I, C^** denotes protected, non-protected, isotype control, and non-antibody treated control animals, respectively.

## Discussion

The results presented here lend strong support to the notion that passively administered bNAb 2G12 is able to offer substantial protection against mucosal SHIV challenge at low serum neutralizing titers. In particular, 3 of 5 macaques showed sterilizing immunity on vaginal challenge with a high dose of SHIV_SF162P3_ when serum-neutralizing titers of 2G12 were of the order of 1∶1. This result contrasts strongly with protection observed with bNAb b12 when sterilizing immunity for the majority of animals is associated with neutralizing titers of very approximately 1∶100 and greater [8],[9],[33]. The result also contrasts with the quantitative studies of Martin and colleagues [10], which show sterilizing immunity against challenge with the ×4 SHIV_DH12_ only at high specific anti-DH12 antibody neutralizing titers. The result is however consistent with studies of Mascola and colleagues who showed that low serum neutralizing titers of 2G12 provided sterilizing immunity for 2 of 4 macaques vaginally challenged with the ×4 virus SHIV_89.6P_ [7].

We investigated factors that might help explain the protective efficacy of 2G12, especially in relation to b12. This efficacy might be explained if 2G12 was transported very effectively to the site of infection; the results presented suggest this is not the case. We next noted that, although the neutralizing titers of 2G12 in our experiments are low, the actual serum concentrations of 2G12 are high since the antibody neutralizes the challenge virus SHIV_SF162P3_, and indeed other available SHIVs rather poorly. Therefore, it is possible that the protective efficacy of 2G12 derives from another anti-viral function of antibody that becomes important at high antibody concentration. One such function could be antibody-mediated host cell activity against SHIV-infected cells, which can be measured in the ADCVI assay. The results showed that 2G12 is somewhat less effective than b12 in the ADCVI assay. However, at the serum concentrations achieved in the passive transfer experiments, 2G12 should be able to promote infected cell killing *in vivo*. Therefore, one possible explanation for the differing relationship between neutralization and protection for b12 and 2G12 against SHIV_SF162P_ challenge is that protection is determined not by neutralizing ability but solely by activity against infected cells. An argument against this explanation is provided by the observation that b12 can still provide substantial protection in the complete absence of Fc receptor function and ADCVI [9]. Furthermore, HIVIG, which tends to mediate effective ADCVI (GL and DNF, unpublished observations) is rather ineffective at protection against SHIV challenge [5],[7]. An explanation more concordant with the totality of data is that both neutralizing and extra-neutralizing activities are important for protection. Fc-mediated extra-neutralizing activities include, not only host cell activities against infected cells, but also those against free virions such as phagocytosis. It may be that 2G12 is able to compensate for its weak neutralization of SHIV_SF162P3_ by effective extra-neutralizing activities such as ADCVI. Protection studies using a SHIV that is sensitive to neutralization at low 2G12 concentrations but only sensitive to extra-neutralizing activities at high concentrations may help to better separate the contributions of different mechanisms to protection here. Alternatively, a 2G12 mutant lacking effector activity analogous to that generated for b12 [9] may help towards this aim.

One intriguing difference between b12 and 2G12 that might relate to differences in protection has been described in terms of neutralization kinetics (PP, unpublished observations). Thus, it appears that following antibody-virus preincubation *in vitro*, neutralization by 2G12 occurs almost immediately while b12-mediated neutralization slowly progresses with time, reaching 2G12 neutralization levels only after hours. Only at high concentrations, corresponding to high neutralization titers, do b12 neutralization kinetics match those of 2G12. *In vivo* such kinetic differences may give 2G12 a noticeable advantage over b12: immediate efficiency at preventing target cell infection may be of particular importance *in vivo* as delay in neutralization may lead to cell infection and viral spread spinning out of antibody control. Further studies looking at correlations between antibody neutralization kinetics and protection efficiency should help reveal whether kinetics are important for protection.

A further consideration for 2G12 is that the antibody recognizes high mannose glycans on the envelope gp120 surface. These glycans are also recognized by a number of lectins including DC-SIGN, which has been proposed to have a critical role in transmission by facilitating the transport of virus by dendritic cells to lymphoid tissues [34],[35]. Indeed, it has been shown that the addition of an N-glycan site to the V2 loop of SF162P leads to a gain of DC-SIGN binding and that this correlates with enhanced mucosal transmission of SHIV_SF162P3_ [36]. The gp120-DC-SIGN interaction can be perturbed by 2G12 but not b12 as shown in a number of assays including inhibition of whole virus binding to DC-SIGN-expressing cell lines [37],[38]. If the HIV-DC-SIGN interaction is critically important for the establishment of infection, then it is possible that 2G12 protection is mediated by inhibiting this interaction. Intriguingly, although 2G12 requires relatively high concentrations to neutralize SHIV_SF162P3_, presumably because of relatively low affinity for the envelope trimer [39],[40],[41], it binds with high nM affinity to monomeric gp120 from SHIV_SF162P3_. HIV is suggested to express both functional and other forms of envelope including monomeric envelope [42],[43]. If DC-SIGN was exploiting nonfunctional as well as or instead of functional envelope on virions then 2G12 might be an efficient competitor for binding to nonfunctional envelope at the *in vivo* concentrations achieved in our passive experiments. Future studies on the potential role of inhibiting the HIV-DC-SIGN interaction in blocking transmission could make use of anti-DC-SIGN antibodies.

In summary, the data presented here, together with earlier data, show conclusively that monoclonal antibody 2G12 can offer protection against mucosal SHIV challenge at low neutralization titers. An explanation based on unusual 2G12 transudation properties to the mucosal surface is ruled out. Viable explanations include: (1) rapid 2G12 neutralization kinetics, (2) a critical role for extra-neutralizing, e.g. Fc-mediated, 2G12 activities under the conditions of the experiment, and (3) a critical role for 2G12 inhibition of virus interaction with lectin, e.g. DC-SIGN, bearing cells. Further *in vivo* protection studies will be required to distinguish these possibilities. Nevertheless, the results are provocative in suggesting the glycan shield as a potentially favorable HIV vaccine target.

## Materials and Methods

### Macaques

All protocols for female Indian rhesus macaques were reviewed and approved by the Institutional Animal Care and Use Committees. The animals were housed in accordance with the American Association for Accreditation of Laboratory Animal Care Standards. At the start of the experiments, all animals were experimentally naïve and were negative for antibodies against HIV-1, SIV, and type D retrovirus. Virus challenge and *i.v.* antibody protocols are more fully described elsewhere [8],[44].

### Challenge virus

The virus used in this study was SHIV_SF162P_ passage 3, which has been described elsewhere [45],[46],[47]. SHIV_SF162P3_ retains the R5 phenotype of HIV-1_SF162_. SHIV_SF162P3_, propagated in phytohemagglutin (PHA)-activated rhesus macaque peripheral blood mononuclear cells (PBMC), was obtained through the NIH AIDS Research and Reference Reagent Program, Division of AIDS, NIAID, NIH (Cat. No. 6526; Contributors: Drs. Janet Harouse, Cecilia Cheng-Mayer, and Ranajit Pal).

### Antibodies

Recombinant 2G12 was obtained from Polymun Scientific, Vienna, Austria. The isotype control antibody DEN3, an anti-Dengue NS1 human IgG1 antibody was expressed in Chinese hamster ovary (CHO-K1) cells in glutamine-free custom formulated Glasgow minimum essential medium (GMEM Selection Media) (MediaTech Cellgro). For large-scale tissue culture, media was supplemented with 3.5% Ultra Low Bovine IgG Fetal Bovine Serum (Invitrogen) and grown in 10-layer Cellstacks and Cell Cubes (Corning). The antibody was purified using Protein A affinity matrix (GE Healthcare), and dialyzed against phosphate-buffered saline (PBS). Care was taken to minimize endotoxin contamination, which was monitored using a quantitative chromagenic Limulus Amoebecyte Lysate assay (Lonza) performed according to the manufacturer's recommendations. Antibody used for the passive transfer experiments contained <1 IU of endotoxin/mg.

### Plasma viral loads

The quantity of SIV viral RNA genomic copy equivalents (vRNA copy Eq/ml) in EDTA-anticoagulated plasma was determined using a quantitative reverse-transcription PCR (QRT-PCR) assay. Briefly, vRNA was isolated from plasma using a GuSCN-based procedure as previously described [48]. QRT-PCR was performed using the SuperScript III Platinum One-Step Quantitative RT-PR System (Invitrogen, Carlsbad, CA). Reaction mixes did not contain bovine serum albumin (BSA). Reactions were run on a Roche Lightcycler 2.0 instrument and software. vRNA copy number was determined using LightCycler 4.0 software (Roche Molecular Diagnostics, Indianapolis, IN) to interpolate sample crossing points onto an internal standard curve prepared from 10-fold serial dilutions of a synthetic RNA transcript representing a conserved region of SIV *gag*.

### Serum antibody ELISAs

2G12 antibody concentrations in macaque sera were determined in ELISA by three different methods: (1) by binding to an immobilized synthetic oligomannose dendron [21] conjugated to BSA; (2) by binding to the anti-Idiotype 2G12 mouse IgG1 L13 kindly provided by Polymun Scientific, Vienna, Austria [49]; and (3) by binding to monomeric gp120 JR-FL kindly provided by Progenics [8].

### Antibody concentrations in vaginal secretions

The determination of antibody concentration in mucosal secretions was performed as described by Kozlowski, et al. [8],[50]. Briefly, vaginal secretions from each animal were absorbed to cellulose wicks (Solan Weck-Cel surgical spears; Xomed Surgical Products, Jacksonville, FL). A set of 3 samples per animal was taken at 6 hours, 12 hours, 24 hours, 4 days, and 7 days post *i.v.* antibody administration. Wicks were weighed before and after secretion absorption. Clarified supernatants extracted from the wicks were used to determine the concentration of antibody in mucosal secretions by ELISA. Resulting data was compared to the corresponding antibody standard curve using nonlinear regression. Arithmetic means and standard deviations were calculated for each set of triplicate samples per animal. The differences in the mean concentrations of b12 and 2G12 at each timepoint were evaluated in a student's t test. Analyses performed in GraphPad Prism Software for Mac, Version 5.0a.

### Neutralization assays

Neutralization of antibodies and sera was assessed by 2 different methods. Neutralization of the primary isolate SHIV_SF162P3_, was performed using phytohemagglutinin (PHA)-activated peripheral blood mononuclear cells (PBMC) from a single rhesus macaque (no. 355) as target cells. Cells from this animal replicate SHIV_SF162P_ efficiently. Neutralization assessment was carried out as described previously [8]. Neutralization titers of animal sera were reported by Monogram Biosciences, South San Francisco, CA after preparation of an HIV-1 envelope pseudotyped luciferase SHIV_SF162P3_ capable of single-round replication. The pseudovirus-based neutralization assay was performed as previously described [51].

### MHC genotyping

MHC genotyping by sequence-specific PCR was performed by the University of Wisconsin Genotyping Core with support of NIH grant 5R24RR16038-6 awarded to David I. Watkins and previously described [25].

### Viral sequence amplification

Viral RNA was extracted from 140 µl of monkey serum using the QIAamp Viral RNA Mini Kit (Qiagen) according to the manufacturer's instructions. 8 µl of this viral RNA was then used for cDNA synthesis using Superscript III (Invitrogen) primed by primer sf162rtn (5′-TTATAGCAAAATCCTTTCC-3′). 3 ul of the cDNA reaction was then used to amplify the gp160 open reading frame using primers sf162mf (5′–CACCATGAGAGTGAAGGGGATCAGGAAG-3′) and sf162rn (5′-TTATAGCAAAATCCTTTCCAAGCCCTGTC-3′) in combination with PfuUltra Hotstart DNA Polymerase from Stratagene. After an initial denaturation step at 95°C for 4 minutes, 35 cycles were performed with 95°C for 30 seconds, 52°C for 30 seconds and 72°C for 3 minutes, before a final elongation at 72°C for 10 minutes concluded the amplification. Sequences were determined after subcloning the PCR products into TOPO vectors.

### Statistics

The experiment consisted of a total of 9 animals (n = 9) divided into treatment groups as follows: 2 animals (n = 2) in the isotope control group, 2 animals (n = 2) in the non-antibody-treated controls, and 5 animals (n = 5) in the 2G12-treated group. Statistical analyses were performed using Graph Pad Prism for Windows, version 5 (Graph Pad Software Inc., San Diego, CA, 2005).

### Protein Sequences

GenBank accession locus for 2G12 is 10M3_H (heavy chain, Fab 2G12 unliganded) and 10M3_L (light chain, Fab 2G12 unliganded). GenBank accession locus for IgG1 b12 is AAB26306.

## References

[1] Johnston MI, Fauci AS (2007). An HIV vaccine–evolving concepts.. N Engl J Med.

[2] Barouch DH (2008). Challenges in the development of an HIV-1 vaccine.. Nature.

[3] Walker BD, Burton DR (2008). Toward an AIDS vaccine.. Science.

[4] Shibata R, Igarashi T, Haigwood N, Buckler-White A, Ogert R (1999). Neutralizing antibody directed against the HIV-1 envelope glycoprotein can completely block HIV-1/SIV chimeric virus infections of macaque monkeys.. Nat Med.

[5] Mascola JR, Lewis MG, Stiegler G, Harris D, VanCott TC (1999). Protection of macaques against pathogenic simian/human immunodeficiency virus 89.6PD by passive transfer of neutralizing antibodies.. J Virol.

[6] Baba TW, Liska V, Hofmann-Lehmann R, Vlasak J, Xu W (2000). Human neutralizing monoclonal antibodies of the IgG1 subtype protect against mucosal simian-human immunodeficiency virus infection.. Nat Med.

[7] Mascola JR, Stiegler G, VanCott TC, Katinger H, Carpenter CB (2000). Protection of macaques against vaginal transmission of a pathogenic HIV-1/SIV chimeric virus by passive infusion of neutralizing antibodies.. Nat Med.

[8] Parren PW, Marx PA, Hessell AJ, Luckay A, Harouse J (2001). Antibody protects macaques against vaginal challenge with a pathogenic R5 simian/human immunodeficiency virus at serum levels giving complete neutralization in vitro.. J Virol.

[9] Hessell AJ, Hangartner L, Hunter M, Havenith CE, Beurskens FJ (2007). Fc receptor but not complement binding is important in antibody protection against HIV.. Nature.

[10] Nishimura Y, Igarashi T, Haigwood N, Sadjadpour R, Plishka RJ (2002). Determination of a statistically valid neutralization titer in plasma that confers protection against simian-human immunodeficiency virus challenge following passive transfer of high-titered neutralizing antibodies.. J Virol.

[11] Parren PW, Ditzel HJ, Gulizia RJ, Binley JM, Barbas CF (1995). Protection against HIV-1 infection in hu-PBL-SCID mice by passive immunization with a neutralizing human monoclonal antibody against the gp120 CD4-binding site.. Aids.

[12] Trkola A, Purtscher M, Muster T, Ballaun C, Buchacher A (1996). Human monoclonal antibody 2G12 defines a distinctive neutralization epitope on the gp120 glycoprotein of human immunodeficiency virus type 1.. J Virol.

[13] Binley JM, Wrin T, Korber B, Zwick MB, Wang M (2004). Comprehensive cross-clade neutralization analysis of a panel of anti-human immunodeficiency virus type 1 monoclonal antibodies.. J Virol.

[14] Li M, Gao F, Mascola JR, Stamatatos L, Polonis VR (2005). Human immunodeficiency virus type 1 env clones from acute and early subtype B infections for standardized assessments of vaccine-elicited neutralizing antibodies.. J Virol.

[15] Sanders RW, Venturi M, Schiffner L, Kalyanaraman R, Katinger H (2002). The mannose-dependent epitope for neutralizing antibody 2G12 on human immunodeficiency virus type 1 glycoprotein gp120.. J Virol.

[16] Scanlan CN, Pantophlet R, Wormald MR, Ollmann Saphire E, Stanfield R (2002). The broadly neutralizing anti-human immunodeficiency virus type 1 antibody 2G12 recognizes a cluster of alpha1–>2 mannose residues on the outer face of gp120.. J Virol.

[17] Calarese DA, Scanlan CN, Zwick MB, Deechongkit S, Mimura Y (2003). Antibody domain exchange is an immunological solution to carbohydrate cluster recognition.. Science.

[18] Calarese DA, Lee HK, Huang CY, Best MD, Astronomo RD (2005). Dissection of the carbohydrate specificity of the broadly neutralizing anti-HIV-1 antibody 2G12.. Proc Natl Acad Sci U S A.

[19] Zanetti G, Briggs JA, Grunewald K, Sattentau QJ, Fuller SD (2006). Cryo-Electron Tomographic Structure of an Immunodeficiency Virus Envelope Complex In Situ.. PLoS Pathog.

[20] Roux KH, Taylor KA (2007). AIDS virus envelope spike structure.. Curr Opin Struct Biol.

[21] Wang SK, Liang PH, Astronomo RD, Hsu TL, Hsieh SL (2008). Targeting the carbohydrates on HIV-1: Interaction of oligomannose dendrons with human monoclonal antibody 2G12 and DC-SIGN.. Proc Natl Acad Sci U S A.

[22] Marx PA, Spira AI, Gettie A, Dailey PJ, Veazey RS (1996). Progesterone implants enhance SIV vaginal transmission and early virus load.. Nat Med.

[23] Roux KH, Zhu P, Seavy M, Katinger H, Kunert R (2004). Electron microscopic and immunochemical analysis of the broadly neutralizing HIV-1-specific, anti-carbohydrate antibody, 2G12.. Mol Immunol.

[24] Yant LJ, Friedrich TC, Johnson RC, May GE, Maness NJ (2006). The high-frequency major histocompatibility complex class I allele Mamu-B*17 is associated with control of simian immunodeficiency virus SIVmac239 replication.. J Virol.

[25] Kaizu M, Borchardt GJ, Glidden CE, Fisk DL, Loffredo JT (2007). Molecular typing of major histocompatibility complex class I alleles in the Indian rhesus macaque which restrict SIV CD8+ T cell epitopes.. Immunogenetics.

[26] Loffredo JT, Maxwell J, Qi Y, Glidden CE, Borchardt GJ (2007). Mamu-B*08-positive macaques control simian immunodeficiency virus replication.. J Virol.

[27] Casimiro DR, Wang F, Schleif WA, Liang X, Zhang ZQ (2005). Attenuation of simian immunodeficiency virus SIVmac239 infection by prophylactic immunization with dna and recombinant adenoviral vaccine vectors expressing Gag.. J Virol.

[28] Pal R, Venzon D, Letvin NL, Santra S, Montefiori DC (2002). ALVAC-SIV-gag-pol-env-based vaccination and macaque major histocompatibility complex class I (A*01) delay simian immunodeficiency virus SIVmac-induced immunodeficiency.. J Virol.

[29] Zhang ZQ, Fu TM, Casimiro DR, Davies ME, Liang X (2002). Mamu-A*01 allele-mediated attenuation of disease progression in simian-human immunodeficiency virus infection.. J Virol.

[30] Mothe BR, Weinfurter J, Wang C, Rehrauer W, Wilson N (2003). Expression of the major histocompatibility complex class I molecule Mamu-A*01 is associated with control of simian immunodeficiency virus SIVmac239 replication.. J Virol.

[31] Yasutomi Y, McAdam SN, Boyson JE, Piekarczyk MS, Watkins DI (1995). A MHC class I B locus allele-restricted simian immunodeficiency virus envelope CTL epitope in rhesus monkeys.. J Immunol.

[32] Su J, Luscher MA, Xiong Y, Rustam T, Amara RR (2005). Novel simian immunodeficiency virus CTL epitopes restricted by MHC class I molecule Mamu-B*01 are highly conserved for long term in DNA/MVA-vaccinated, SHIV-challenged rhesus macaques.. Int Immunol.

[33] Gauduin MC, Parren PW, Weir R, Barbas CF, Burton DR (1997). Passive immunization with a human monoclonal antibody protects hu-PBL-SCID mice against challenge by primary isolates of HIV-1.. Nat Med.

[34] Geijtenbeek TB, Kwon DS, Torensma R, van Vliet SJ, van Duijnhoven GC (2000). DC-SIGN, a dendritic cell-specific HIV-1-binding protein that enhances trans-infection of T cells.. Cell.

[35] Soilleux EJ, Morris LS, Leslie G, Chehimi J, Luo Q (2002). Constitutive and induced expression of DC-SIGN on dendritic cell and macrophage subpopulations in situ and in vitro.. J Leukoc Biol.

[36] Lue J, Hsu M, Yang D, Marx P, Chen Z (2002). Addition of a single gp120 glycan confers increased binding to dendritic cell-specific ICAM-3-grabbing nonintegrin and neutralization escape to human immunodeficiency virus type 1.. J Virol.

[37] Binley JM, Ngo-Abdalla S, Moore P, Bobardt M, Chatterji U (2006). Inhibition of HIV Env binding to cellular receptors by monoclonal antibody 2G12 as probed by Fc-tagged gp120.. Retrovirology.

[38] Hong PW, Nguyen S, Young S, Su SV, Lee B (2007). Identification of the optimal DC-SIGN binding site on human immunodeficiency virus type 1 gp120.. J Virol.

[39] Roben P, Moore JP, Thali M, Sodroski J, Barbas CF (1994). Recognition properties of a panel of human recombinant Fab fragments to the CD4 binding site of gp120 that show differing abilities to neutralize human immunodeficiency virus type 1.. J Virol.

[40] Sattentau QJ, Moore JP (1995). Human immunodeficiency virus type 1 neutralization is determined by epitope exposure on the gp120 oligomer.. J Exp Med.

[41] Parren PW, Wang M, Trkola A, Binley JM, Purtscher M (1998). Antibody neutralization-resistant primary isolates of human immunodeficiency virus type 1.. J Virol.

[42] Poignard P, Moulard M, Golez E, Vivona V, Franti M (2003). Heterogeneity of envelope molecules expressed on primary human immunodeficiency virus type 1 particles as probed by the binding of neutralizing and nonneutralizing antibodies.. J Virol.

[43] Moore PL, Crooks ET, Porter L, Zhu P, Cayanan CS (2006). Nature of nonfunctional envelope proteins on the surface of human immunodeficiency virus type 1.. J Virol.

[44] Veazey RS, Shattock RJ, Pope M, Kirijan JC, Jones J (2003). Prevention of virus transmission to macaque monkeys by a vaginally applied monoclonal antibody to HIV-1 gp120.. Nat Med.

[45] Harouse JM, Gettie A, Eshetu T, Tan RC, Bohm R (2001). Mucosal transmission and induction of simian AIDS by CCR5-specific simian/human immunodeficiency virus SHIV(SF162P3).. J Virol.

[46] Harouse JM, Gettie A, Tan RC, Blanchard J, Cheng-Mayer C (1999). Distinct pathogenic sequela in rhesus macaques infected with CCR5 or CXCR4 utilizing SHIVs.. Science.

[47] Tan RC, Harouse JM, Gettie A, Cheng-Mayer C (1999). In vivo adaptation of SHIV(SF162): chimeric virus expressing a NSI, CCR5-specific envelope protein.. J Med Primatol.

[48] Cline AN, Bess JW, Piatak M, Lifson JD (2005). Highly sensitive SIV plasma viral load assay: practical considerations, realistic performance expectations, and application to reverse engineering of vaccines for AIDS.. J Med Primatol.

[49] Wolbank S, Kunert R, Stiegler G, Katinger H (2003). Characterization of human class-switched polymeric (immunoglobulin M [IgM] and IgA) anti-human immunodeficiency virus type 1 antibodies 2F5 and 2G12.. J Virol.

[50] Kozlowski PA, Cu-Uvin S, Neutra MR, Flanigan TP (1999). Mucosal vaccination strategies for women.. J Infect Dis.

[51] Richman DD, Wrin T, Little SJ, Petropoulos CJ (2003). Rapid evolution of the neutralizing antibody response to HIV type 1 infection.. Proc Natl Acad Sci U S A.

[52] Friedrich TC, Valentine LE, Yant LJ, Rakasz EG, Piaskowski SM (2007). Subdominant CD8+ T-cell responses are involved in durable control of AIDS virus replication.. J Virol.

